# DNA Image Cytometry for Screening the Carcinogenetic Risk of Oral Potential Malignant Disorders

**DOI:** 10.7150/jca.91048

**Published:** 2024-01-01

**Authors:** Huan Li, Wenhui Li, Yijun Gao, Junqi Li, Xiliang Zeng, Jiaming Lin, Xiaoyan Xie, Tianyou Ling

**Affiliations:** 1 Department of Stomatology, The Second Xiangya Hospital, Central South University, 139 Renmin Middle Road, Changsha 410011, People's Republic of China.; 2 Changsha JianLu Medical Laboratory Co, 18 Luyun Road, Changsha 410006, People's Republic of China.

**Keywords:** DNA-Image Cytometry, DNA aneuploidy, Oral submucosal fibrosis, Oral Leukoplakia, oral potentially malignant disorder, epithelial dysplasia

## Abstract

**Background:** Oral Submucosal Fibrosis (OSF) and Oral Leukoplakia (OLK) are well-known oral potentially malignant disorders, and cases of Oral Submucosal Fibrosis concomitant Oral Leukoplakia (OSF+OLK) are now being reported clinically. DNA image cytometry is an objective and non-invasive method for monitoring the risk of precancerous lesions in the oral cavity.

**Methods:** A total of 111 patients with clinically characterized oral mucosal lesions underwent simultaneous and independent histopathological and DNA imaging cytometry assessments. Clinical data were also collected for each patient.

**Results:** The frequency of DNA content abnormality was higher in the tongue than in other oral sites (P = 0.003) for OLK. The frequency of DNA content abnormality was higher in the tongue than in other oral sites (P = 0.035) for OSF+OLK. The differences of DNA content abnormality in age, sex, dietary habit, smoking, and alcohol intake were not observed in OLK and OSF+OLK. The study indicates an association between DNA content abnormality and pathological examination in OSF+OLK ( χ^2^ test, P = 0.007). OLK showed higher sensitivity and specificity than OSF, while the sensitivity and specificity of OSF+OLK are higher than OLK only and OSF only.

**Conclusion:** DNA image cytometry can be utilized as an adjunctive device for the initial detection of oral potentially malignant disorders that require further clinical management.

## Introduction

Oral submucosal fibrosis (OSF) is an insidious chronic oral disease that is commonly found in South and East Asian countries such as India, Pakistan, Bangladesh, Nepal, Sri Lanka, China, and the Pacific Islands, where the disease is closely related to the culture of betel nut chewing^1^. The main clinical manifestations include blanching and hardening of the oral mucosa, pain when eating irritating foods, and progressive mouth opening limitation, which seriously affects the patient's quality of life. According to the World Health Organization, there are more than 5 million OSF patients worldwide. In addition, OSF is broadly regarded as an oral potentially malignant disorder (OPMD). Previous studies have reported that the global malignancy rate of OSF varied from 1.2% to 23%^2^.

Oral Leukoplakia (OLK) is defined as a white plaque of questionable risk having excluded (other) known diseases or disorders that carry no increased risk for cancer and is considered the most frequently encountered and typical OPMD^3^, ^4^. Variable proportions of malignant transformation have been reported in different studies, ranging from 0.13% to 34.0%^5^.

Several articles have mentioned a phenomenon often observed in clinical settings, where OSF cases may be accompanied by OLK^6^-^9^. According to the reports, the incidence of OSF concomitant OLK (OSF+OLK) was 4.8%-24.5% in OSF cases. Notably, the malignancy rate of OSF + OLK (11.1%-18.5%) was significantly higher than that of OSF only (4.6-7.2%) and OLK only (4.7%) in Taiwan, China^10^. This implies that OSF concomitant OLK is an important OPMD that deserves increased awareness by clinicians. However, it is difficult for clinicians to adequately identify oral precancerous lesions by visual inspection and palpation, and even when patients are clinically diagnosed, it is still hard to assess the level of risk of the disease. This result in oral cancer usually not being diagnosed until advanced stages, rather than at the precancerous stage, when the cancer cells have become aggressive^11^. Therefore, early detection and diagnosis of oral precancerous lesions and oral cancer is quite critical and may have a great impact on improving survival rates.

Currently, epithelial dysplasia is usually considered as a method to discriminate the malignant potential of OPMD. However, it is uncertain whether incisional biopsies from suspected OPMD are representative of the histologic results of the whole lesion^12^. In addition, this method is not acceptable to many patients due to its invasiveness, especially when the lesion is present in a seemingly "normal" or asymptomatic oral mucosa. Furthermore, invasive serial biopsies have limited reproducibility when performing long-term monitoring of patients with suspected oral lesions^13^. Other than that, it is time-consuming, operator- and pathologist-dependent, and used primarily in hospitals. Hence, painless, noninvasive, and objective diagnostic techniques are needed for the early detection of dysplasia within OPMD and to help monitor the progression of OPMD.

DNA aneuploidy is considered the cytometric equivalent of chromosomal aneuploidy and is an internationally recognized marker of neoplastic transformation in lung, cervical, and liver diseases as well as oral diseases^14^-^19^. DNA-Image Cytometry (DNA-ICM) on Feulgen-stained oral mucosal cell smears can be used as an early diagnostic test for the malignant transformation of squamous epithelial cells^20^. In this blinded prospective study on OSF and OLK and OSF+OLK, the aim of this study was to examine the correlation between DNA content and clinical parameters and epithelial dysplasia of the oral mucosa, as well as to assess the role of DNA image cytometry in the diagnosis of epithelial dysplasia in different patient groups.

## Materials and methods

### Patients and the sample

This study was approved by the Institutional Review Board of The Second XiangYa Hospital of Central South University [Approval No.2019SK2124] and written informed consent was obtained from all subjects. In this study, patients with clinical components of OLK or OSF and OSF+OLK lesions who visited the dental clinic at the Department of Oral Mucosal Diseases, Second XiangYa Hospital, Central South University, were prospectively enrolled from October 2020 to December 2021. The clinical data collected included age, sex, smoking, alcohol intake, dietary habit, and lesion site.

The clinician instructs the patient to rinse the mouth with water and then gently scrape the oral mucosal lesions at least 10 times using the flexible cotton cytology brush. The brush head was stored in a tube with cell preservation solution. Brush samples were then sent to the JianLu Medical Laboratory within 24 hours for DNA quantitative analysis. These samples were examined using a liquid-based thin-layer cell preparation and Feulgen staining. Incisional biopsies were performed at the same locations where brush samples were obtained, and the biopsies were fixed in formaldehyde solution and embedded in paraffin. Histological diagnosis of dysplasia was performed by two pathologists from the pathology department of our hospital according to the World Health Organization (WHO) guidelines^21^.

### DNA-image cytometry and Diagnostic Criteria

The samples were deposited on microscope slides and the slides were stained with the Feulgen-thionin technique, which stains the nuclei dark blue without staining the cytoplasm, and subsequently scanned with the GEMINI Medical Diagnostic System's (Changsha) DNA Image Cytometer (DNA-ICM). The DNA content of the cells is expressed according to c (content), with G1/G0 cells being 2c cells (diploid cells) and G2/M cells being 4c cells (tetraploid cells). There are usually no aneuploid cells except in the S phase where the cell DNA content is between 2c and 4c. However, when carcinogenesis occurs, aneuploid cells appear. DNA quantitative analysis was performed only when specimens contained at least 300 epithelial cell nuclei that were normalized to normal epithelial cell nuclei. DNA image cytometry was performed blinded to the histological results. The resulting DNA index (DI) is the ratio of the DNA content of the analyzed sample nuclei to the reference normal nuclear DNA content and is used to classify epithelial nuclei into groups: DNA diploid group has a DI of 0.75-1.25, DNA aneuploid (DNA index≥ 2.3), hyperdiploid group(1.25 ≥DNA index ≥ 1.75), and DNA tetraploid (1.25≥ DNA index ≥2.3). According to the criteria for evaluation of the previous reports and ESACP guideline, if the aneuploid group has more than 3 nuclei (1% of at least 300 nuclei), or the hyperdiploid group had 5% of the total number of nuclei, or the tetraploid group had 10% of the total number of nuclei, the oral specimen was classified as having abnormal DNA content^22^-^25^.

### Histologic examination

Histopathological sections were independently detected by two pathologists who were blinded to the DNA-ICM results. According to the WHO definition of OLK, white lesions with the respective clinical manifestations and pathological features are excluded, such as leukoedema, linea alba, chronic biting irritation, white spongy nevus, and other potentially malignant disorders such as discoid lupus erythematosus and lichen planus. The pathology of OSF is characterized by hypercollagenous deposits in the subepithelial connective tissue of the oral mucosa and local inflammation of the lamina propria or deep connective tissue, as well as muscle degenerative changes^26^. In addition, epithelial cell atrophy, and loss of rete pegs have been reported^27^.

### Results evaluation and analysis

SPSS 26.0 software package was used for statistical analysis of the data. Chi-square test or Fisher's Exact test were used to evaluate the differences between qualitative variables. We calculated the sensitivity, specificity, positive and negative predictive values (PPV and NPV) with 95%CI of quantitative DNA analysis in the diagnosis of epithelial dysplasia in different oral mucosal lesions, referring to the histopathological diagnosis as the gold standard. All tests were two-sided, with P<0.05 as the level of significance.

## Results

### Correlation between DNA content and clinicopathological features of OSF and OLK and OSF+OLK

**Figure 1:**
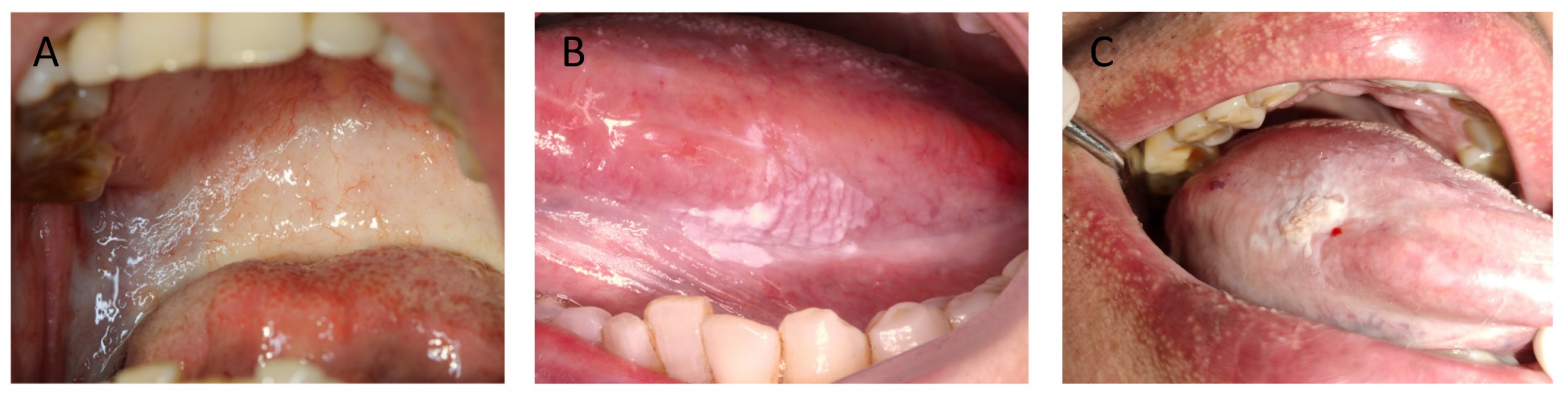
A, a case of OSF presents as the soft and pink oral mucosa appear pale with horizontal bands across the soft palate. B, a case of OLK presents as white plaques on the lateral margin of the tongue. C, a case of OSF + OLK presents as the presence of white plaques on the lateral margin of the blanched tongue.

A total of 111 patients with a clinical and pathologic diagnosis of OSF or OLK or OSF+OLK were enrolled in this study, Figure 1 illustrates clinical presentations of three lesions. 333.3% patients of OSF were evaluated for DNA content abnormality, 50% patients of OLK were evaluated for DNA content abnormality, 60.00% patients of OSF+OLK were were evaluated for DNA content abnormality. The life habits and pathologic features of these patients are showed in Table 1. The frequency of DNA content abnormality was higher in tongue (68.00%) than in other oral sites (9.09%, χ^2^ test, P = 0.003) for OLK. The frequency of DNA content abnormality was higher in tongue (78.95%) than in other oral sites (46.15%, χ^2^ test, P = 0.035) for OSF+OLK. The differences of DNA content abnormality in age, sex, dietary habit, smoking, alcohol intake were not observed in OSF, OLK and OSF+OLK. The study indicates an association between DNA content abnormality and pathological examination in OSF+OLK (χ^2^ test, P = 0.007).

### Cytological diagnoses in comparison with histopathological diagnoses in different patient groups (Table 2)

**Figure 2:**
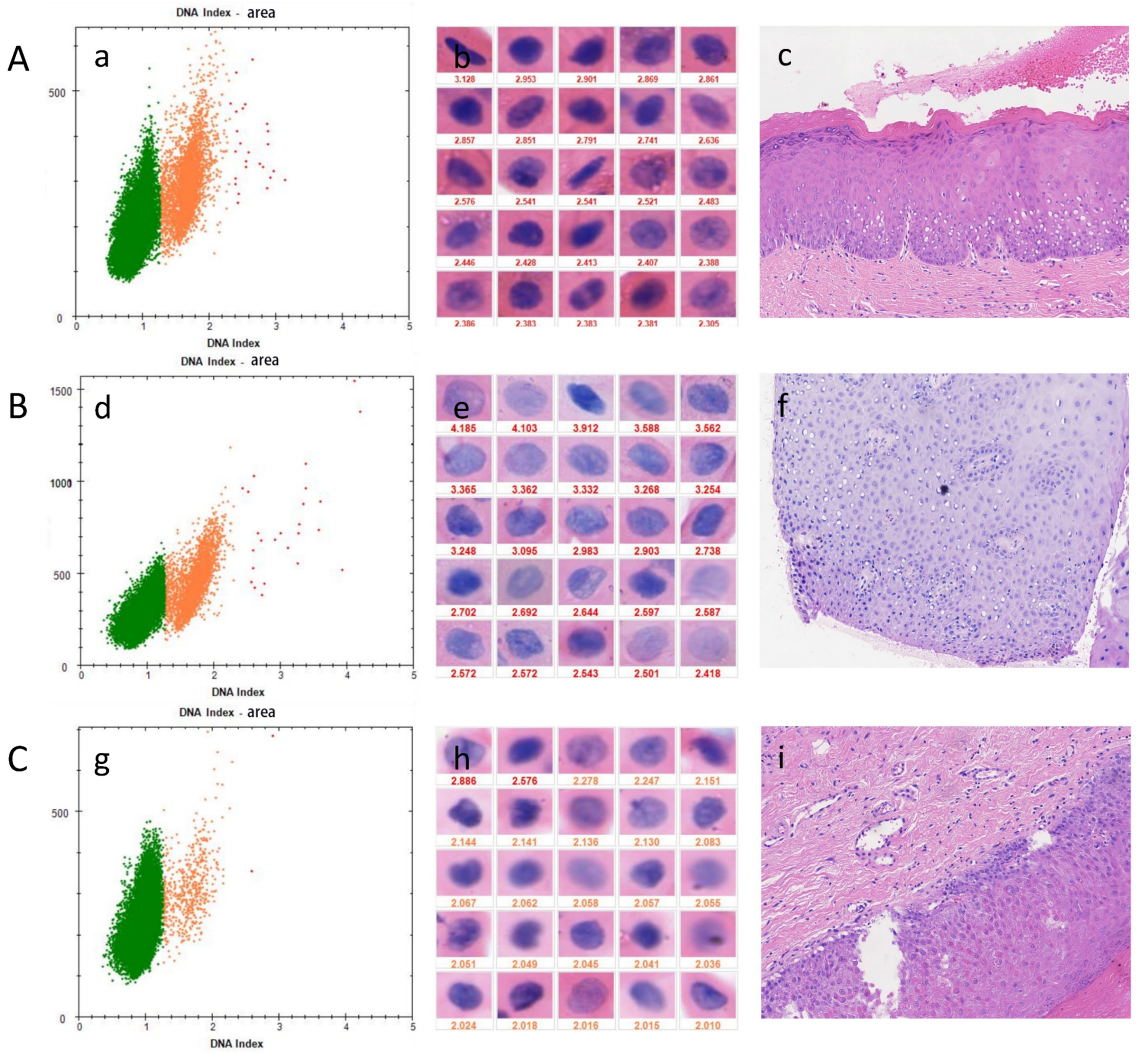
DNA-ICM and histopathology of three cases of OPMD. A, a case of OLK lesion with more than 3 nuclei DI values ≥ 2.3 was diagnosed as mild-dysplasia. B, a case of OSF lesions more than 3 nuclei DI values ≥ 2.3 was diagnosed as mild-dysplasia. C, A case of OSF+OLK lesion with less than 3 nuclei DI values ≥ 2.3 was diagnosed as non-dysplasia. (a), (d), (g) the scatter diagram shows the number of cells with different DI;(b), (e), (h) the morphology of aneuploidy cells and normal cells (stained Feulgen, lens × 400). (Staining Feulgen, lens ×400); (c), (f), (i) HE stains of Biopsy tissue (lens × 100)

For different types of oral lesions (Table 3), the sensitivity of OSF+OLK with dysplasia was 79.17% while the specificity was 61.90%. The sensitivity of OSF with dysplasia was 57.14% while the specificity was 73.91%. OLK with dysplasia showed a sensitivity of 58.33% and specificity of 66.67%. Presence of dysplasia showed a sensitivity of 67.27% and specificity of 67.86% (Table 3). Figure 2 shows the examination results for three different types of diseases.

**Figure 3:**
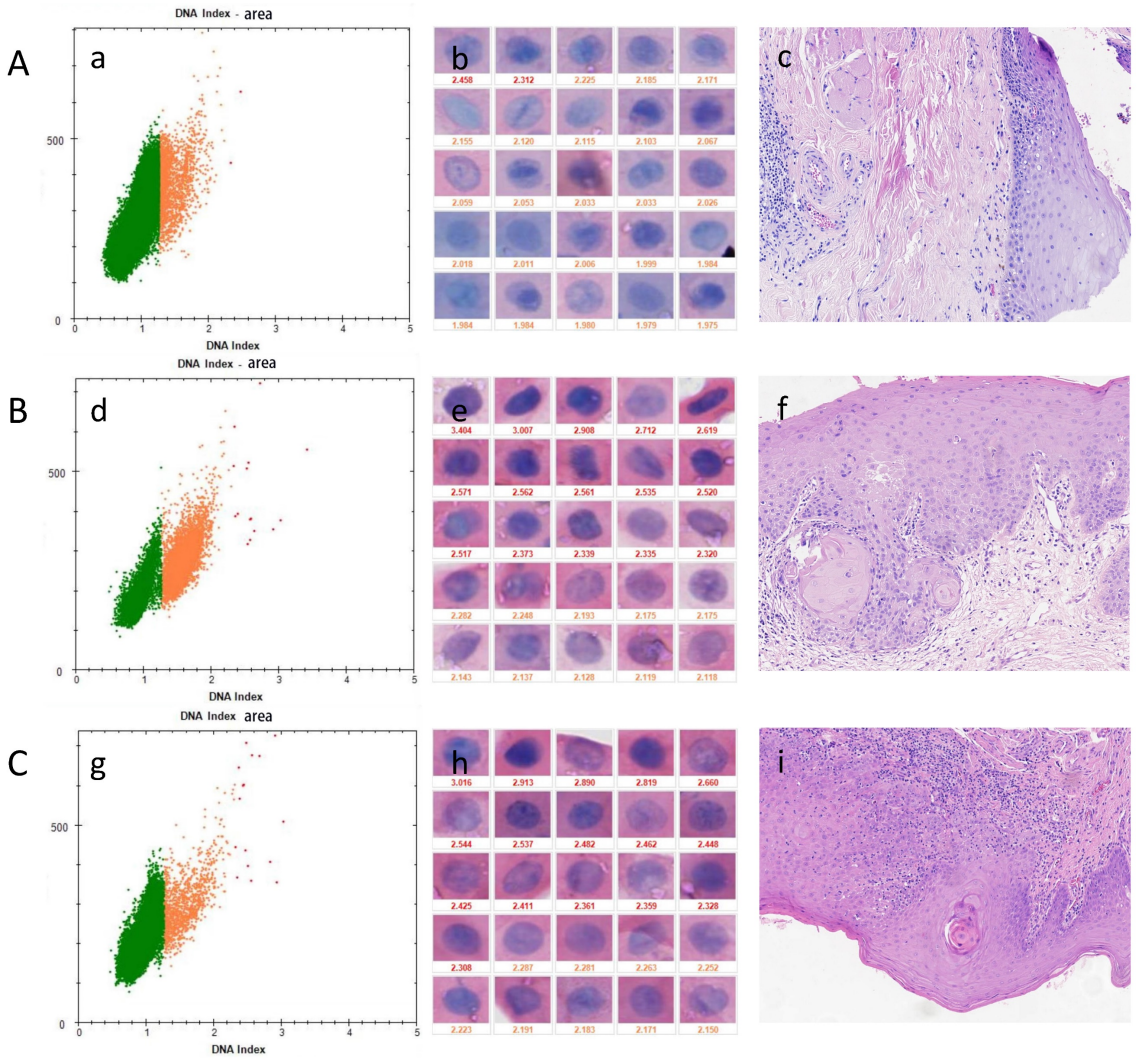
DNA-ICM and histopathology of three cases of OPMD. A, a case of OLK lesion with less than 3 nuclei DI ≥ 2.3 was diagnosed as mild-dysplasia. B, a case of OSF+OLK lesion more than 3 nuclei DI ≥ 2.3 was diagnosed as moderate-dysplasia. C, a case of OSF lesion with more than 3 nuclei DI ≥ 2.3 was diagnosed as severe-dysplasia. A was a false-negative representative case. (a), (d), (g) the scatter diagram shows the number of cells with different DI; (b), (e), (h) the morphology of aneuploidy cells and normal cells (stained Feulgen, lens × 400). (Staining Feulgen, lens ×400); (c), (f), (i) HE stains of Biopsy tissue (lens × 100)

In all types of oral lesions (Table 4), the sensitivity of detecting mild dysplasia was59.09% while the specificity was 67.86%. The sensitivity of detecting moderate dysplasia was 68.97% while the specificity was 67.86%. Severe dysplasia showed a sensitivity of 100.0% and specificity of 67.86%. The presence of dysplasia showed a sensitivity of 67.27% and specificity of 67.86%. The sensitivity increased as the grade of epithelial dysplasia increased. Figure 3 shows three cases of OPMDs with different levels of epithelial dysplasia, and A was a false-negative representative case.

**Figure 4:**
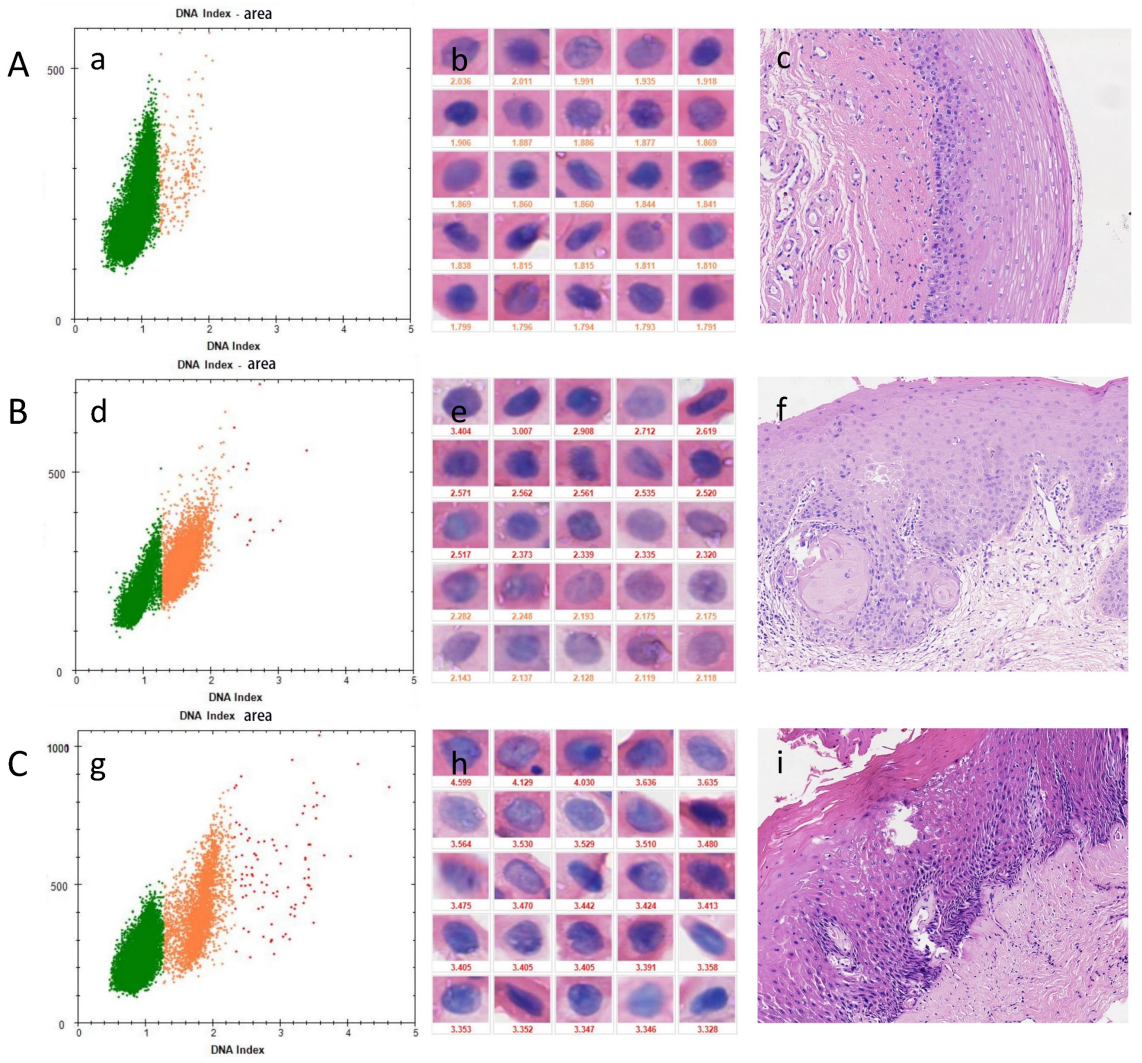
DNA-ICM and histopathology of three cases of OSF+OLK. A, a case of OSF+OLK lesion with no nuclei DI ≥ 2.3 was diagnosed as mild-dysplasia. B, a case of OSF+OLK lesion more than 3 nuclei DI ≥ 2.3 was diagnosed as moderate-dysplasia. C, a case of OSF + OLK lesion with more than 3 nuclei DI ≥ 2.3 was diagnosed as severe-dysplasia. (a), (d), (g) the scatter diagram shows the number of cells with different DI; (b), (e), (h) the morphology of aneuploidy cells and normal cells (stained Feulgen, lens × 400). (Staining Feulgen, lens × 400); (c), (f), (i) HE stains of Biopsy tissue (lens × 100).

In OSF+OLK (Table 5), the sensitivity of detecting mild dysplasia was 70.00% while the specificity was 61.90%. The sensitivity of detecting moderate dysplasia was 81.82% while the specificity was 61.90%. Severe dysplasia showed a sensitivity of 100.0% and specificity of 61.90%. Presence of dysplasia showed a sensitivity of 79.16% and specificity of 61.90%. The sensitivity increased as the grade of epithelial dysplasia of OSF+OLK increased. Three positive cases are shown in Figure 4.

## Discussion

Oral cancer may develop from oral potentially malignant disease (OPMD). OSF is a recognized OPMD with a high rate of progression to OSCC and OLK is the best known potentially malignant disease of oral cancer with a higher malignancy rate than OSF. It has been clinically observed that OSF may complicate OLK, and it was demonstrated that OLK could enhance the rate of malignant transformation from 7.2% of OSF only to 15.2% of it concomitant OLK, which is an important type of OPMDs^7^. Currently, dysplasia is recognized as a risk factor for assessing malignant transformation of OPMDs, but non-dysplastic OPMDs (ND-OPMDs) have also been found to develop oral cancer^28^. Therefore, predicting the risk of OPMD progression to carcinoma remains a challenge and requires a painless, noninvasive and objective early adjuvant diagnosis.

DNA aneuploidy is an indicator of chromosomal changes, the presence of which is often a critical early warning step in carcinogenesis^29^. In addition, the hypothesis of DNA aneuploidy as a marker to predict oral carcinogenesis has tremendous clinical implications. Although DNA aneuploidy cytology may represent a potential noninvasive adjunctive diagnostic tool for the early detection of oral carcinogenesis, previous studies have reported broad sensitivity (16.0%-96.4%) and specificity (66.6%-100%) of DNA-ICM in screening for OPMD using tooth brushing, which may be due to variations in disease type^30^. When oral cancer cases are included, the sensitivity and specificity of DNA-ICM are significantly improved.

In this study, each participant underwent brush biopsy and scalpel biopsy and histological examination at the same lesion site of the oral lesion. No differences in DNA content were observed with respect to age, gender, dietary habits, smoking, or alcohol intake, contrary to our previous clinical knowledge. The current preliminary study reported that the site of OLK and OSF+OLK lesion occurrence (p<0.05) was significantly associated with abnormal DNA content^25^. Therefore, scalpel biopsy and histopathological examination are routinely recommended in order to monitor patients for OLK and OSF+OLK lesions occurring in the tongue. Our data show that the risk of abnormal DNA content is much higher in patients with OLK occurring in the tongue, so we recommend aggressive treatment of tongue leukoplakia, such as surgical excision or laser excision. However, our preliminary data show that DNA content status does not correlate with the presence of dysplasia in OLK and OSF+OLK, which is inconsistent with some earlier findings and may be related to cytologic alterations of the lesion that precede histologic alterations^31^. Because non-invasive DNA image cytometry is readily available and has a high level of patient acceptance., more high-risk OSF+OLK and OLK lesions can be screened and monitored, and therefore more oral cancers may be detected at an early or precancerous stage of development. In addition, as mentioned earlier, oral mucosa without abnormal epithelial hyperplasia can also become cancerous, which may be related to the fact that oral mucosal disease is usually large in size, so pathological biopsies may not always be taken accurately to the site with abnormal epithelial hyperplasia. Brush biopsies with DNA content analysis can take cells from the entire lesion and help to monitor such oral lesions over time.

Previous study reported that 41.5%-48.6% of OLK patients were identified with abnormal DNA content, similar to our findings (50.0%). A lower percentage of OSF patients (33.3%) were identified with abnormal DNA content than OLK. Alarmingly, 60.0% of OSF+OLK patients were identified with abnormal DNA content. It is reasonable to speculate that OLK may augment DNA content abnormalities and lesion progression in OSF, which is consistent with the finding that OLK can enhance the rate of malignant transformation.

OLK shows higher sensitivity and specificity than OSF, since the histopathological features of OSF are mainly epithelial atrophy with a limited number of epithelial cells and dysplasia grading does not work well for OSF. In addition, OSF most frequently occurs in the buccal mucosa, retromolar area, and the soft palate sites, and incisional biopsy of suspicious lesions of OSF is difficult to represent the overall lesion^32^, ^33^. However, our study reported that the sensitivity and specificity of OSF+OLK was higher than that of OLK only and OSF only. This result may be due to the fact that most OSF have only less dysplasia and due to atrophy and textural hardening of the oral mucosal epithelium. And the keratinized surface of OLK is probably responsible for a large number of false negative results because cell preparation from brushings can be inadequate and is limited to the superficial or intermediate layers of the oral mucosa making it difficult to detect dysplastic changes. Positive smears were obtained from lesions with epithelial atypia which have nonkeratinized or ulcerated but not keratinized surfaces which occurs to OSF+OLK frequently in our study.

Besides, in the current study, the sensitivity and specificity increased as the grade of epithelial dysplasia increased, which proved that abnormal DNA content can help to detect high-risk diseases. Our study have shown that DNA-ICM exhibits 100% sensitivity in severe epithelial dysplasia, but it is probably associated with the limited number of cases.

In addition, we should note that we reported false-positive and negative cases in our study: the false-positive rate was higher for mild abnormal epithelial hyperplasia. Previous studies have reported that DNA-cytology diagnosis of malignancy performed approximately 1-15 months before histological diagnosis may explain the false-positive diagnosis^19^, ^34^. Overlapping exfoliated cells on smears can also lead to false positives. Inflammatory exudates and necrotic disintegrants can interfere with the procedure of obtaining exfoliated cells, leading to false-negative rates. Therefore, we should avoid obtaining exfoliated cells at this stage of the disease. Initial processing of both brush biopsies and scalpel biopsies is good when inflammatory exudate or necrotic disintegration is present in potentially malignant oral disease. We must try to reduce the rate of false positives and false negatives by (a) there should be a good distribution of exfoliated cells on the smear and (b) the examination should be performed after the exudate and crusts have subsided.

Other factors that influence the success of DNA-ICM include the type of brush, sample preparation and staining, cellularity, and the use of automated scanners to accurately measure cellular DNA content with minimal human intervention^35^. The cotton brushes we used in this study may have resulted in reduced accuracy due to the inability to obtain cells from the entire epithelial layer of the oral mucosa.

The limitation of our study is that it is a cross-sectional diagnostic study, and we will further conduct adequate long-term follow-up at a later stage to assess the effectiveness of this procedure as a predictive strategy for malignant transformation of OPMD. DNA-ICM should also be studied in combination with other noninvasive techniques (e.g., TCT, microRNA, autofluorescence imaging, and toluidine blue staining) to improve detection results. DNA-ICM can also help screen for lesions in a community setting, thereby reducing unnecessary biopsies and improving detection of high-risk oral diseases. It is important to note that the use of this technology is not intended to replace biopsies in clinical workups, but rather for initial screening of oral mucosal disease, especially as it can be used in community and private practice screenings to help clinicians identify lesions that require further examination. This will allow for timely intervention of high-risk lesions, thereby improving patient survival.

## Conclusion

DNA image cytometry can be utilized as an adjunctive device for the initial detection of oral potentially malignant disorders that require further clinical management.

## Figures and Tables

**Table 1:** Correlation between DNA content and clinicopathological features of 111 patients with OSF and OLK and OSF+OLK

Characteristic n(%)	OSF			OLK			OSF+OLK		
	DNA content		P	DNA content		P	DNA content		P
	Abnormal	Normal		Abnormal	Normal		Abnormal	Normal	
Patients	10	20		18	18		27	18	
Age (years)									
Mean	37.1	36.6		45	44		46.4	38.9	
Range	30-52	25-59		39-51	36-51		32-60	30-60	
Sex									
Female	1(10.00)	0(0.00)	0.333	0(0.00)	0(0.00)	NA	0(0.00)	1(5.56)	0.400
Male	9(90.00)	20(100.00)		18(100.00)	18(100.00)		27(100.00)	17(94.44)	
Dietary habit									
Bland	4(40.00)	7(35.00.)	1.000	10(55.56)	11(61.11)	1.00	13(48.15)	13(72.22)	0.134
Spicy	6(60.00)	13(65.00)		8(44.44)	7(38.89)		14(51.85)	5(27.78)	
Smoking									
Never	2(20.00)	4(20.00)	1.000	5(27.78)	7(38.89)	0.725	6(22.22)	2(11.11.)	0.445
Past and present	8(80.00)	16(80.00)		13(72.22)	11(61.11)		21(77.78)	16(88.89)	
Alcohol intake									
Never	9(90.00)	17(85.00)	1.000	13(72.22)	12(66.67)	1.00	26(96.30)	17(94.44)	1.00
Past and present	1(10.00)	3(15.00)		5(27.78)	6(33.33)		1(3.70)	1(5.56)	
Lesion site									
Nontongue				1(5.56)	10(55.56)	0.003	12(44.44)	14(77.78)	0.035
Tongue				17(94.44)	8(44.44)		15(55.56)	4(22.22)	
Epithelial dysplasia									
No	6(60.00)	17(85.00)	0.181	4(22.22)	8(44.44)	0.289	8(29.63)	13(72.22)	0.007
Mild or moderate or severe	4(40.00)	3(15.00)		14(77.78)	10(55.56)		19(70.37)	5(27.78)	

† NA, not available

**Table 2:** Results of cytological diagnoses in comparison with histopathological diagnoses

	Epithelial dysplasia	DNA		Total
		Abnormal	Normal	
**OSF+OLK**	No	8	13	21
	Mild	7	3	10
	Moderate	9	2	11
	Severe	3	0	3
	Total	27	18	45
**OSF**	No	6	17	23
	Mild	3	3	6
	Severe	1	0	1
	Total	10	20	30
**OLK**	No	4	8	12
	Mild	3	3	6
	Moderate	11	7	18
	Total	18	18	36

**Table 3:** Measurement of DNA content for the diagnosis of epithelial dysplasia in different types of oral lesions

	Lesion	Sensitivity(95%CI)	Specificity(95%CI)	PPV(95%CI)	NPV(95%CI)
**OPMDs**	OSF+OLK	79.17%(57.29%~92.06%)	61.90%(38.69%~81.05%)	70.37%(49.66%~89.29%)	72.22%(46.41%~89.29%)
	OSF	57.14%(20.24%~88.19%)	73.91%(51.31%~88.92)	40.00%(13.69%~72.63%)	85.00%(61.14%~96.04%)
	OLK	58.33%(36.94%~77.20%)	66.67%(35.44%~88.73%)	77.78%(51.92%~92.63%)	44.44%(22.40%~68.65%)
	Total	67.27%(53.18%~78.95%)	67.86%(53.91%~79.35%)	67.27%(53.18%~78.95%)	67.86%(53.91%~79.35%)

**Table 4:** Measurement of DNA content for the diagnosis of mild, moderate, and severe epithelial dysplasia in OSF+OLK and OSF and OLK

	Epithelial dysplasia	Sensitivity(95%CI)	Specificity(95%CI)	PPV(95%CI)	NPV(95%CI)
**OPMDs**	Mild	59.09%(36.68%~78.52%)	67.86%(53.91%~79.35%)	41.94%(25.07%~60.74%)	80.85%(66.27%~90.35%)
	Moderate	68.97%(49.05%~84.02%)	67.86%(53.91%~79.35%)	52.63%(36.05%~68.69%)	80.85%(66.27%~90.35%)
	Severe	100.0%(39.58%~100.00%)	67.86%(53.91%~79.35%)	18.18%(5.99%~41.00%)	100.0%(88.57%~100.00%)
	Total	67.27%(53.18%~78.95%)	67.86%(53.91%~79.35%)	67.27%(53.18%~78.85%)	67.86%(53.91%~79.35%)

**Table 5:** Measurement of DNA content for the diagnosis of mild, moderate and severe epithelial dysplasia in OSF+OLK

	Epithelial dysplasia	Sensitivity(95%CI)	Specificity(95%CI)	PPV(95%CI)	NPV(95%CI)
**OSF+OLK**	Mild	70.00%(35.37%~91.91%)	61.90%(38.69%~81.05%)	46.67%(22.28~72.58%)	81.25%(53.69%~95.03%)
	Moderate	81.82%(47.76%~96.77%)	61.90%(38.69%~81.05%)	52.94%(28.53%~76.14%)	86.67%(58.39%~97.66%)
	Severe	100.0%(31.00%~100.00%)	61.90%(38.69%~81.05%)	27.27%(7.33%~60.68%)	100.00%(71.66%~100.00%)
	Total	79.16%(57.29%~92.06%)	61.90%(38.69%~81.05%)	70.37%(49.66%~85.50%)	72.22%(46.41%~89.29%)
